# Part-of-Speech tagging enhancement to natural language processing for Thai wh-question classification with deep learning^☆^

**DOI:** 10.1016/j.heliyon.2021.e08216

**Published:** 2021-10-19

**Authors:** Saranlita Chotirat, Phayung Meesad

**Affiliations:** a Department of Information Technology, Faculty of Information Technology and Digital Innovation, King Mongkut's University of Technology North Bangkok, Thailand; b Department of Information Technology Management, Faculty of Information Technology and Digital Innovation, King Mongkut's University of Technology North Bangkok, Thailand

**Keywords:** Question classification, Thai sentence analysis, Part-of-Speech (POS) tag, Feature selection

## Abstract

Question classification is a crucial task for answer selection. Question classification could help define the structure of question sentences generated by features extraction from a sentence, such as who, when, where, and how. In this paper, we proposed a methodology to improve question classification from texts by using feature selection and word embedding techniques. We conducted several experiments to evaluate the performance of the proposed methodology using two different datasets (TREC-6 dataset and Thai sentence dataset) with term frequency and combined term frequency-inverse document frequency including Unigram, Unigram+Bigram, and Unigram + Trigram as features. Machine learning models based on traditional and deep learning classifiers were used. The traditional classification models were Multinomial Naïve Bayes, Logistic Regression, and Support Vector Machine. The deep learning techniques were Bidirectional Long Short-Term Memory (BiLSTM), Convolutional Neural Networks (CNN), and Hybrid model, which combined CNN and BiLSTM model. The experiment results showed that our methodology based on Part-of-Speech (POS) tagging was the best to improve question classification accuracy. The classifying question categories achieved with average micro $F_{1}$-score of 0.98 when applied SVM model on adding all POS tags in the TREC-6 dataset. The highest average micro $F_{1}$-score achieved 0.8 when applied GloVe by using CNN model on adding focusing tags in the Thai sentences dataset.

## 1 Introduction

In recent years, we have required large amounts of information to retrieve the answer via the question answering applications. Questioning is a significant ability in both human and intelligent engines. Questioning is the key to gaining more information and is very useful in many applications. We use the questioning ability to ask for information or seeking answers. The desirable answers could be explored in many resources such as textbooks, encyclopedias, Wikipedia, etc. While readers seeking an answer will need to deal much more deeply with the problem of extracting the meaning of a text in a rich sense. Readers always seek to find an answer based on the type of question encountered. Because question and corresponding answers are related depending on question types, the readers can answer the question based on a keyword. Using words with the same meaning in the question is complicated to train a text model to understand language like humans.

Question classification (QC) is an essential part in many applications [1], such as Question Answering (QA) [2], [3], Information Retrieval (IR), E-learning systems, and Question generation [4]. Question classification learns matching questions to one class or multiple classes and helps identify text's answer types. Question classification has two main approaches [5]. The first one is a manual classification by handmade rules to identify expected answer types. The second one is an automatic classification. The wh-questions would help the reader identify the information. For example, “Who” can identify the characters of the narrative story, “When” can identify the time happening, and “Where” can identify the location. Therefore, if the readers understand how to use wh-questions, they can improve their reading comprehension.

Most studies are focused on question classification in English; however, there are some research works on other languages such as Chinese [6], Arabic [7], Indonesian [4], and Thai language [8]. It is an early stage for the Thai language in this area. The study of Thai texts is challenging due to some aspects because of the limitation of accuracy in analyzing text classification in Thai texts that convey a different meaning when considered alone than when joining other words because of a meaning word based on ordering the sequence of words and context. Thus, analyzing the context of words is necessary and could improve by applying Natural Language Processing tasks for sentence tokenization and defining each term in sentences. Recent literature shows that many research works successfully combine Natural Language Processing with preprocessing techniques to gain better machine learning features. Part-of-Speech (POS) tagging is part of the popular feature selection method based on definition and context, i.e., Noun, Verb, Adjective, etc. However, a detailed assessment of the question classification and question generation from a simple sentence is missing in Thai texts. Thus, this is a research opportunity.

In this paper, we proposed a methodology for feature selection method based on a POS tag category for questing answering models. We compared the efficiency of the proposed method with classification models that classify sentences in the question category. We used machine learning models based on Multinomial Naïve Bayes (MNB), Logistic Regression (LR), Support Vector Machine (SVM). Besides, Bidirectional Long Short-Term Memory (BiLSTM), Convolutional Neural Network (CNN), and Hybrid model (combined CNN with BiLSTM model) were also employed.

We organize the rest of this paper as follows. In Section 2, we start with the literature reviews and related works. We show the research methodology in Section 3. Section 4 gives details of experimental results. Finally, we gave concluding remarks in section 5.

## 2 Related work

### 2.1 Question classification

The purpose of text classification is to select one or more classes for unstructured textual data, depending on the content of the text. Many popular applications apply text classification in Information Retrieval (IR) and Question Answering Systems (QAS), such as sentiment analysis, topic classification, and question classification. Sentiment analysis is concerned with analyzing texts to the sentiments or emotions/opinions. Sentiment analysis defines a word/multi-word that conducts a Positive, Neutral, or Negative sentiment. The topic classification based on keyword extraction determines the correlation between each classified topic and the given article. Then it assigns the most related article as the possibly selected topic.

The question classification task defines one or several categories of the entity (or answer) type from the passage. Question classification is a necessary process of question answering and question generation intelligence that requires the system to retrieve answers or generate questions based on that type of question such as factoid, definition, and list [5]. Different question labels were proposed in [9]. The authors in [9] defined a two-layered taxonomy, which represents a natural semantic classification for typical answers in the TREC task. The hierarchy contains six coarse classes (Abbreviation, Entity, Description, Human, Location, and Numeric) and 50 fine classes.

In this research, we defined the question classification to wh-question as relating to persons, time, location, and measurements.

Who: Words that are a name of a person or organization.

Where: Words that represent locations, such as market, city, and university.

When: Words that describe the date and time.

How: Words represent the counted elements, determiner, and measurements.

### 2.2 Text preprocessing

Natural Language Processing enables computers to understand natural language as humans do. Text preprocessing is a traditionally important step for Natural Language Processing. There are several steps in text preprocessing. It starts with text cleaning in which the incomplete and noisy data are removed before passing them into a classifier. Next, words tokenization, punctuation removal, abbreviation expansion, stop words removal, common words removal, Named Entities Recognition (NER), and Part-of-Speech (POS) tagging are performed in a pipeline. Part-of-Speech (POS) tagging is known as entity extraction for extracting features and marks the word in a text with labels including Nouns, Verb, Adverb, and so on, in that context.

The literature reviews about applied text preprocessing on text classification and question classification show that text preprocessing is a key for success classification accuracy. The authors in [10] studied the impact of data preprocessing for prediction review ratings. They compared the different methods for preprocessing. The results showed that removing common words, lower-casing, simple stop word elimination, and a combination of n-grams improve performance classification on review rating stars. The authors in [11] studied the effect of POS tagging to enhance Arabic text classification performance by focusing on certain POS tags. They showed that using Nouns, Verbs, and Adjectives as features could increase performance while the words could achieve better accuracy when fewer features were. The authors in [12] considered the effect of POS tags on Arabic text classification by considering only POS tags as nouns and adjectives. They stated that not all word forms affect the meaning of documents. For example, nouns have the sense effectively while adverbs have not to effect. Thus, we have investigated study the impact of occurring words based on the POS tags category.

In [13], the authors mentioned that failure in the POS tagger might affect the performance of the question classification. If a model uses a perfect POS tagger and a flawless feature extracting method, it can classify text with high performance.

Especially, analyzing Thai texts is made difficult by features. The Thai language is complex because it includes adjustable word order, serial verbs, and high incertitude in compound words [14]. It requires a high-performance word segmentation as it has no word boundary indicators to separate words in sentences. The Thai sentences rely on individual judgment when there are unknown words, while English sentences have natural spaces. Besides, Thai has structural ambiguities in which Part-of-Speech (POS) tags relied on context words that affect one word may have the opposite meaning [8], [14], [15], [16], [17]. Thus, data preprocessing has involved the analysis of Thai sentences. In addition, POS tagging could help improve performance for the study of Thai texts. The most probable Part-of-Speech (POS) sequence aims to help determine the type of space for generating questions from Thai text proposed in [8].

### 2.3 Feature selection

Question Classification is the process that can be assorted and defined to accept a question to one or several in the category based on its expected answer type. Previous studies have confirmed that feature selection is helpful for question classification. In 2019, the authors in [1] surveyed studies directly and involved in question classification. They found 88.75% used some extraction/selection mechanism on automatic question classifiers. Many researchers used the feature-based method where features related to a specific subset of questions from the texts before passing into a classifier [2], [16], [18], [19], [20], [21]. Various techniques are dealing with feature selection for improving the performance of question classification.

– Lexical feature represents the relationship between words extracted from the question. The lexical feature considers the frequency of the tokens (or words) with bag-of-words features or N-gram features. Unigram is the most popular used set of lexical features for text classification. Unigram takes a sentence and looks at all the tokens in that sentence. Bigrams, Trigrams, and N-grams look at the sets of consecutive (with n=2, n=3, and n=N, respectively) words in the sentence.

– Syntactic feature represents syntax-related features extracted from the syntactical structure including question headwords and Part-of-Speech (POS) tags. Headword is considered from the keywords or key phrases which contain important information for the sense to answer. Analyzes the sentences in many common words that may have several meanings. Each word in the sentence was marked by its Part-of-Speech in the text preprocessing task. POS is popular essential element in Natural Language Processing for extracting features and marks the word in a text with labels. The function of a POS tagger is to solve the deficiencies based on the context of words. They are known as entity extraction for identifying words as nouns, verbs, and adverbs.

– Semantic feature: Semantic features concern the meaning of words. It is based on the semantic meaning of the words by requires a dictionary or WordNet to extract semantic information. The semantic feature could recognize from words that often assign to objects, actions, and properties. Named entity recognizer is popularly used to provide semantic information in text classification that predefined semantic category for a noun. For example, Named Entity Recognition (NER) is used to classify nouns into different semantic categories such as a person, organization, location, and time.

Three main features techniques [21] were implemented for question classification. (1) Grammatical feature consists of a word class (Noun, Verb, Adverb, Adjective, Determiner, Conjunction, and Preposition), a subclass of the word (Common Nouns, Proper Nouns, Action Verb, Linking Verb, etc.), and question words (Who, Where, When, What, and How). (2) Domain-specific features are related to the target of question-answering or specific term. (3) Grammatical pattern features consider the sequence of occurring words which different pattern representation benefits recognizing the diverse question type. The authors in [2], [21] used a grammatical feature and syntactic categories related to different types that improved the classification of the factoid question type. [22] developed a question answering system improving the answer selection process by using POS-tagger-based Question Pattern analysis for identified question type for conversation agents, compared to lexical features, syntax features, and semantic features.

Although lexical features are simpler to extract but combined with syntax features, semantic features. Dealing with all types of features can lead to an increase in the performance of classifies. According to [2], [18], [20], [21], the experimental results showed that combining lexical features and syntax features can significantly improve the accuracy of classifying. The experimental results in [20], [23] showed that combining Unigram with N-gram features outperformed others for classification. [20] proposed data preprocessing applied Part-of-Speech tag features, and combined number of n-grams can increase the accuracy of the text classification. The authors in [24] studied N-gram and embedding features with the word, POS tags, and mixed features for native language identification. They found that the results of combined Unigram and Tri-gram features could improve the best accuracy while no significant improvement when using N-gram more than three.

Unigram or Bag-of-words (BOW) and Inverse Document Frequency (TF-IDF) are the main techniques for feature extraction and selection [1]. There are advantages to using N-gram in text classification. Many researchers seek the best-fitted combination of term frequency-inverse document frequency (TF-IDF) and N-grams for different data sizes. [25] investigated the impact on feature extraction methods for sentiment classification from user reviews. They applied Unigram, Bigram, and Trigram as N-gram vectorization models with TF-IDF features extraction approaches. The results showed that bigram with TF-IDF and Trigram with TF-IDF yielded better accuracy with a large dataset. Given this viewpoint found from literature, applying these feature techniques in Thai texts would lead to better performance of Thai question classification.

There are few studies about using feature techniques for analysis and classification texts in the Thai language. [16] studied sentiment analysis in Thai texts by combining word embedding, Part-of-Speech tags, and semantic features and fusing deep learning algorithms. Their experimental results showed that combining the features could improve sentiment analysis in Thai texts. [14] suggested the methods to increase the performance of the Thai sentiment classification with points to add weighing scheme for several POS, add disambiguate word senses, add negation into the process, and improve the Thai sentiment resource. This paper was inspired by authors in [16], [20], [21] as more compacts set of syntactic and lexical features combine studied grammatical pattern features for objective to affect the performance of question classification.

### 2.4 Question classification algorithms

Question classification can be divided into two main types include manual classification method and automatic method [5]. Moreover, question classification could divide into approaches based on their model architectures. There are three main approaches including Rule-based, Machine-based, and Hybrid-based approaches [7]. We focus on machine learning approaches for question classification. There are two main machine-based approaches for question classification including traditional methods and emerging deep learning methods.

In this paper, we experiment by using three standard algorithms of traditional methods (Multinomial Naïve Bayes, Logistic Regression, and Support Vector Machines) and three approaches of deep learning model (Bidirectional Long Short-Term Memory, Convolutional Neural Networks, and Hybrid model).

Multinomial Naïve Bayes (MNB) is an advanced version of Naïve Bayes for text classification. The idea is to calculate the statistical frequency of each feature or word in classes to define the possibility of a sentence belonging to a particular category. Support Vector Machine (SVM) is one of the most popular algorithms with high performance for text classification and question classification. SVM used the vectors approach to classifying related documents [1], [26]. Logistic Regression (LR) is a method of statistically analyzing data used to understand the relationship between variables, thereby presenting suitable for general binary classification [20], [26].

Some researchers compared the performance of machine-based approaches for text classification and question classification. SVM techniques provide more efficiency for text classification and question classification [26], [27]. The authors in [27] studied classifying wh-Question types from Arabic texts by using SVM and MNB to classify Questions. Their result has shown that the SVM model is higher performance results than the MNB model. Similarities [26] studied classifying text from English document by compared the accuracy of classifier include SVM, MNB, and LR. They found that the SVM outperforms the rest of the machine-based approaches. The authors in [28] proposed classification on the chatbot application, used the NB method and compared it with the LR method to determine the class intention. The experiment results show the Logistic Regression model is a higher score than the Naïve Bayes model.

Deep learning approaches including Bidirectional Long Short-Term Memory (BiLSTM) and Convolutional Neural Networks (CNN) have emerged as a fulfill tool for classification with higher performance. Bidirectional Long Short-Term Memory (BiLSTM) is one of the recurrent neural network (RNN) models. It efficiently uses past sequence words (via forwarding direction) and future sequence words (via backward direction) for a specific time frame suitable for a natural language processing task. Convolutional Neural Networks (CNN) is a particular feed-forward neural network performed on several tasks such as image classification and natural language processing. CNN has powerful learning ability due to multiple feature extraction stages that can automatically learn representations from the data.

In [29], the authors tried to improve the performance of question answering system using convolutional neural networks and a bidirectional LSTM networks model. Their results showed that the CNN model could learn faster than another model. LSTM network can get better performance, but they use long training time. In contrast, the BiLSTM network seems to have a severe overfitting behavior. The researchers also used CNN-BiLSTM that combined CNN and BiLSTM to improve accuracy. The experimental results showed that the CNN-BiLSTM model improves the text encoding dependencies of the context well. Some other researchers also proposed a hybrid model to enhance the accuracy of text classification. The authors in [17] proposed hybrid model that combined BLSTM with CNN (BLSTM-CNN, BLSTM+CNN, CNN-BLSTM, and BLSTM x CNN) testing with sentiment dataset for comparisons. The experimental results showed that BLSTM-CNN achieved the best performance of sentiment analysis on Thai Texts. Besides, the authors in [30] used a hybrid model based on LSTM and CNN for text classification. They claimed that the proposed hybrid attention LSTM+CNN model had higher accuracy classification performance than a single CNN or LSTM alone.

## 3 Methodology

The literature survey shows an interesting point that there is a need to include the feature selection process for text classification in data preparation. In this research, we use Part-of-Speech (POS) tags or words in conjunction with Thai language sentences. This will helps overcome the limitation of question classification.

**Figure 1:**
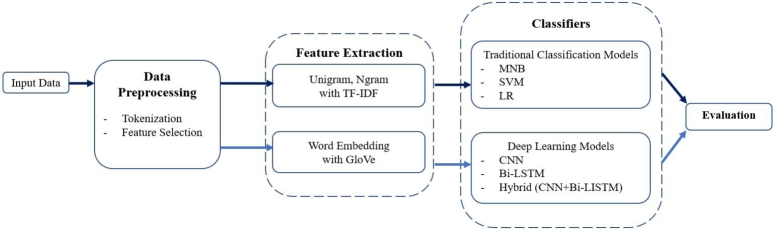
Flowchart of the question classification in these experiments.

The methodology in this research is shown in Fig. 1, comprising five parts: 1) input data, 2) data preprocessing, 3) feature selection, 4) classification, and 5) evaluation. The details of each are described as follows.

### 3.1 Datasets

For performance evaluation of the proposed technique in this research, we used two datasets, the TREC-6 dataset and the Thai sentences dataset.

The first dataset was The Sixth Text REtrieval Conference (TREC-6) [31]. TREC-6 was a short factoid question developed by Li and Roth in [9] that including six coarse question classes (abbreviation, entity, numeric, location, description, and human). TREC-6 consists of two separate sets of 5,452 (training set) and 500 (independent test set). In this research, we split the dataset into a training set of 5,000 samples, a validation set of 452 samples, and a test set of 500 samples.

The second one was Thai sentences collected from Thai Wikipedia. The Thai sentences were manually categorized into wh-question categories: Who, Where, When, and How many. We randomly split the Thai sentence dataset into a training, a validation, and a test dataset. There were 564 training samples, 137 validation samples, and 179 test samples.

### 3.2 Data preprocessing

In this research, we prepared input using data preprocessing with two main steps: tokenization and feature selection.

#### 3.2.1 Tokenization

Thai texts do not have boundary indicators to separate words, not the same as English. Thus, tokenization is a significant phase of data preprocessing. We applied the tokenization method using dictionary-based word segmentation combined with the DeepCut model [32].

#### 3.2.2 Feature selection

Based on our hypothesis, considering POS tags might impact question type, i.e., a noun could refer to location or person. The number could refer to date or volume.

For example “” /mâe gam-lang paa lôok bpai rohng riian/ (A mother is taking a child to the school.) If we consider a sentence with only keywords, it is not easy to define the question type. In addition, if we disregard the syntax features and word sequence, this sentence could be confusing and seen as identical to the question with a different question type.

The noun in the sentence above could refer to “Who” ( /krai /) and “Where” category (/têe năi/) depending on keywords and syntax features. So the probability of question category can be considered as follows: (1) / krai gam-lang paa lôok bpai rohng riian/(Who is taking the child to the school?), (2)  /mâe gam-lang paa lôok bpai têe năi/(Where is the mother taking the child?).

Moreover, some Thai words could have various meanings relying on the sequence of words in a sentence. The meaning of “/gam-lang/” its meaning is “power or energy (Noun),” its meaning is “being (Auxiliary verb),” its meaning is “in the process of (Preposition)”. Therefore, considering a syntactic feature for the obvious classification of Thai sentences is necessary.

In this research, to define the Part-of-Speech (POS) tags, we applied NLP packages including NLP toolkit for Thai (PyThaiNLP) [33] and Stanford CoreNLP [34] for TREC-6 dataset. Then, we considered the frequency of each POS category in question types. The frequency ranked of POS tag occurred in the TREC-6 dataset is shown in Table 1 and Thai sentences dataset is shown in Table 2.

**Table 1:** The rank of the occurrence POS tag on TREC-6.

Rank	Part of Speech tag	Examples
1	NN (Noun, sing or mass)	Llama
2	NNP (Proper noun, Sing.)	IBM
3	JJ (Adjective)	yellow
4	IN (Preposition/sub-conj)	of, in, by
5	DT (Determiner)	a, the
6	VB (Verb base form)	eat
7	NNS (Noun, pular or mass)	llamas
8	VBZ (Verb 3sg pres)	eats
9	VBD (Verb past tense)	ate
10	VBN (Verb past participle)	eaten
11	CD (Cardinal number)	One, two
12	NNPS (Proper noun, plural)	Carolinas

**Table 2:** The rank of the occurrence POS tag on Thai sentences.

Rank	Part of Speech tag	Examples
1	NCMN (Common noun)	(book) /năng-sěu/
2	RPRE (Preposition)	/bon/(on)
3	VSTA (Stative verb)	/keu/(is, are)
4	VACT (Active verb)	/dern/(walk)
5	NPRP (Proper noun)	/koh-roh-nâa/(corona)
6	DCNM (Determiner cardinal number expression)	2 /sŏrng lâym/(two books)
7	NCNM (Cardinal number)	1, /nèung/(one)
8	JSBR (Subordinating conjunction)	/prór wâa/(because)
9	CNIT (Unit classifier)	/chin/(piece)
10	VATT (Attributive verb)	/sŭuay/(beautiful)
11	CMTR (Measurement classifier)	/gì-loh máyt/(Kilometer)
12	CFQC (Frequency classifier)	/kráng/(times)

**Figure 2:**
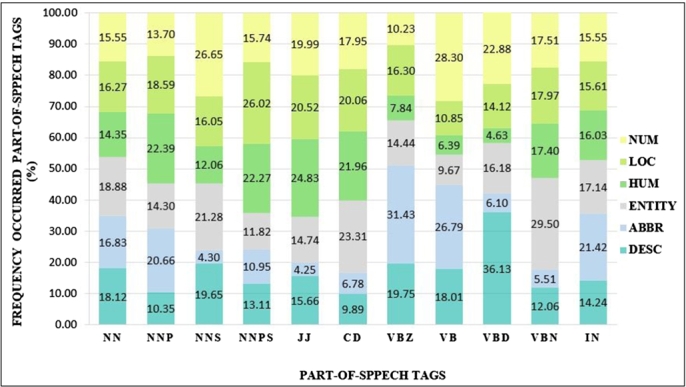
The ratio of POS tag categories on the TREC-6 dataset.

**Figure 3:**
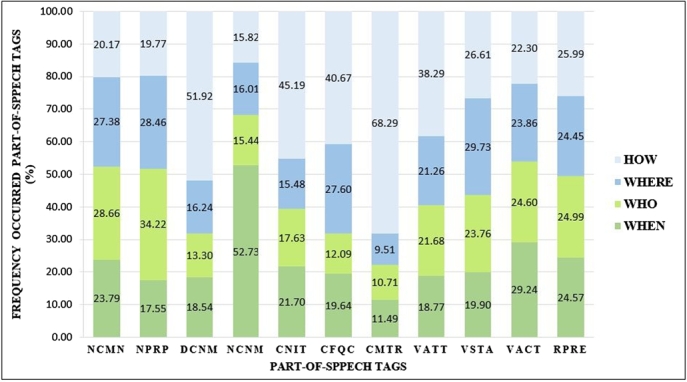
The ratio of POS tag categories on the Thai sentences dataset.

We found that nouns are the most frequently occurring in both TREC-6 and Thai sentences datasets. Besides, we noticed that each question category was sensitive to some features. Thus, we considered the different ratios of occurrence on Part-of-Speech Tags in sentences. The frequency of the “CD (Cardinal number)” tag appeared most often in the Entity class on the TREC-6 dataset, as shown in Fig. 2. Similarly, the “Measurement classifier (CMTR)” tag occurred most often in the “How” class on the Thai sentences dataset, as shown in Fig. 3.

The set of POS tags were dependent on NLP toolkits. We chose POS tags that could make sense to identify the question class and different ratios in each category. Thus, we selected Nouns, Verbs, Adjective, Prepositions, and Determiner, as shown in Table 3.

**Table 3:** Comparisons of related chosen POS tag on TREC-6 and Thai sentences dataset.

Type	Focusing Part-of-Speech tags	
	TREC-6	Thai sentences
Nouns (N)	NN, NNS, NNP, NNPS	NCMN, NPRP
Verbs (V)	VB, VBZ, VBD, VBN	VSTA, VACT
Prepositions (Prep)	IN	RPRE
Adjective (Adj)	JJ	VATT
Determiner (Det)	CD	NCNM, DCNM, CMTR, CFQC, CNIT

We proposed a methodology to improve the question classification performance by focusing on the effect of the POS tags. We considered the top-rank of the occurrence POS tags as focusing POS tags into three feature POS tags sets.

(1) Considering the top-ranked of the occurrence POS tags as focusing POS tags: Nouns, Verbs, Adjectives, Determiner, and Prepositions (N+V+Adj+Dt+Prep: NVADP);
(2) Considering the top-ranked of the occurrence POS tags and ignore Prepositions as focusing POS tags: Nouns, Verbs Adjectives, and Determiner (N+V+Adj+Dt: NVAD);
(3) Considering the top-ranked occurrence POS tags and ignore Verbs and Prepositions as focusing POS tags: Nouns, Adjectives, and Determiner (N+Adj+Dt: NAD).

**Figure 4:**
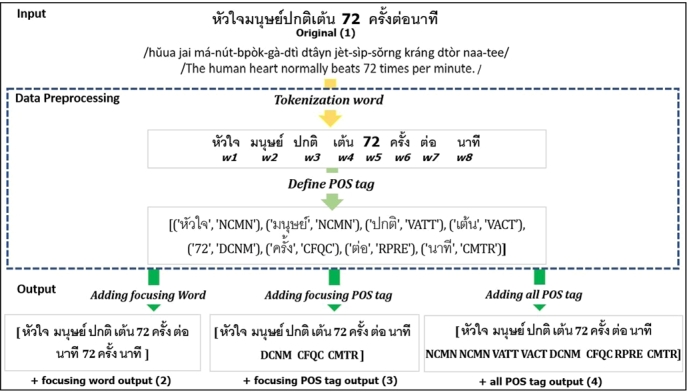
An example of data preprocessing of our proposed method.

To judge the assumption on the effect of selection features to classify the sentences to a question category. We performed several experiments with both the TREC-6 dataset and the Thai sentence dataset to prove concept of the proposed methods. We prepared the input data with four patterns using focusing POS chosen from the previous step as in Algorithm 1, Algorithm 2, and Algorithm 3. Our proposed method is depicted as shown in Fig. 4.

**Algorithm 1:**
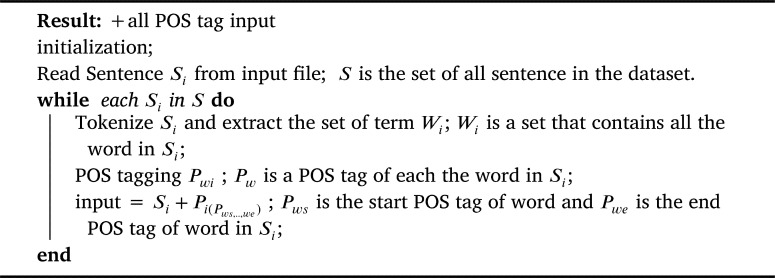
+all POS tag input.

**Algorithm 2:**
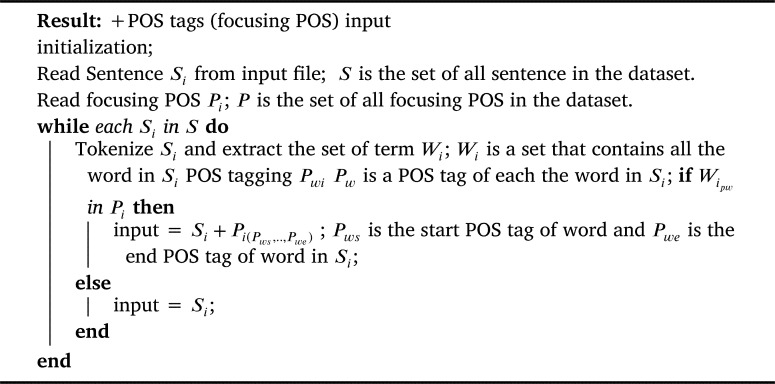
+POS tags input.

**Algorithm 3:**
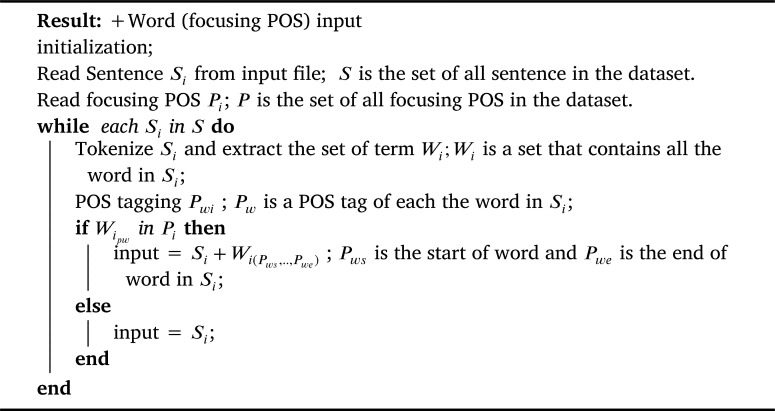
+Word (focusing POS) input.

(1) Original input data were sentences or interrogative sentences from experimental datasets. The formula given a sequence of tokens was $\langle w_{1},w_{2},...,w_{N}\rangle$.

(2) +all POS tags were added all POS tags in original input to output a list of tuples <Pps,Ppe>, each of which was a POS tag in *ps* and *pe*, as presented in Algorithm 1.

(3) +POS tags (focusing POS) were considered adding only POS tags that was focusing POS category mentioned in original input to output a list of tuples <Pps,Ppe>, each of which was only POS tag mentioned in *ps* and *pe*. Here, ps∈[1,N] and pe∈[1,N] were the starting and the ending indexes of a POS tags focused, as presented in Algorithm 2.

(4) +Words (focusing POS) were considered adding words which focusing POS tags in original input to output a list of tuples <Pws,Pwe>, each of which was only words that were POS category mentioned in *ws* and *we*. Here, ws∈[1,N] and we∈[1,N] were the starting and the ending indexes of word in POS tag focused, as presented in Algorithm 3.

### 3.3 Feature extraction

Word embedding vectors have become an effective tool for word representation with continuous vectors. The feature reduction can improve performance by using embedded words. In this research, different from others, we used two datasets consisting of the TREC-6 and Thai sentences datasets. As we mentioned, classifiers will consider sets of *n* words (N-gram).

To find the best combination, we also used term frequency and different variants of term frequency-inverse document frequency including unigram (1-1 gram) with TF-IDF, unigram + bigram (1-2 gram) with TF-IDF, unigram + trigram (1-3 gram) with TF-IDF, bigram (2-2 gram) with TF-IDF, and trigram (3-3 gram) with TF-IDF as features on classification by traditional models. Moreover, we converted each input into sequences of words. We set token inputs of equal length before using them for deep learning.

We applied the post padding on all of the remaining sentences with value zero so that all sentence input had the same length as the longest one with 100 tokens. Nevertheless, if the sequence length was more significant than the maximum length sequence, it was truncated with the max lengths tokens. Then, we used the word embedding from pre-trained GloVe (The Global Vectors for word representation). GloVe was introduced by [35] as an efficient method for learning vector representation of words. It uses count-based methods to capture global statistics by examining words co-occurrence matrix $X_{i,j}$ within a huge texts corpus. The dimension of the glove has embedding vector sizes of 50, 100, 200, and 300 dimensions. We applied pre-trained words by GloVe and the embedded size set to 300. For the TREC-6 dataset. We experimented with GloVe tokens in 6B, 300 dimensions. We used a pre-trained word embedding model trained on Thai Wikipedia data.

### 3.4 Classification

In this work, we used well-known classification models: the traditional model and the deep learning models. Multinomial Naïve Bayes (MNB), Logistic Regression (LR), and Support Vector Machine (SVM) were among the traditional models. The deep learning models were CNN, BiLSTM, and the Hybrid model.

In constructing the CNN model, we used a 1D convolutional layer to convolve the embedding vector with a kernel size of 24 and 32 output filters. Next, we used a ReLU activation function and MaxPooling with a pool size of 24. Next, a Dense layer with a dropout rate of 0.3, and the Softmax layer.

For BiLSTM Model, we constructed with 32 BiLSTM cells followed by Flatten layer, a time distribution dense layer, and a SoftMax layer. We used 32 neurons in the Dense layer and the Softmax layer with four neurons.

In the hybrid model, we used a 1D convolutional layer sliding windows to get the window vector with a kernel of size 2 and 32 output filters followed by a ReLU activation function. We used maximum pooling, input it into BiLSTM, used the Flatten layer for transform vector, and followed by a hidden and dense layer of 32 neurons. Finally, we used a dense layer of 4 neurons with Softmax classification to predict the possible question class as output.

We used the dropout layer to prevent overfitting. We chose the dropout rate from 0.1, 0.2, and 0.3. We found that it was the best performance when we applied with a dropout rate is 0.3. We set 30 epochs for a maximum training time and patience equal to 5 for early stopping. The optimizer used in this experiment was Adam with a learning rate of 2e-3 and batch size of 32.

## 4 Evaluation

In order to evaluate our proposed method, we conducted several experiments by setting each model to run ten times for both datasets. We used accuracy (*A*), precision (*P*), recall (*R*), and $F_{1}$-Score ($F_{1}$) as the performance evaluation metrics as in (1) to (4). The accuracy measures how well the classifier can correctly classify the data into classes. The precision measures the ratio that the classifier can correctly classify the data to a class concerning the total number of data used to test. The recall measures the ratio that the classifier correctly classifies the data concerning the total number of data classified to the class. $F_{1}$-score considers by computing a balance of both precision and recall.

(1) $$A=\frac{TP+TN}{TP+TN+FP+FN}$$

(2) $$P=\frac{TP}{TP+FP}$$

(3) $$R=\frac{TP}{TP+FN}$$

(4) $$F_{1}=2\frac{P\times R}{P+R}$$

where *TP* represents true-positive that is the number of positive samples predicted correctly; *TN* represents true-negative, the number of negative samples predicted correctly; *FP* represents false-positive, the number of negative samples predicted wrongly to positive; and *FN* represents false-negative, the number of positive samples predicted wrongly to negative.

## 5 Experimental results

In the proposed approach, we studied the effect of using POS tags on datasets for comparing the various data preprocessing tasks described in the previous part including (1) Words (Original sentences), (2) Words added POS tags with every word (+all POS Tags), (3) Words added POS tags with considered the focusing POS tags, and (4) Words added words with considered focusing POS tags.

We tested our experiments with different classification models. The selected techniques were Multinomial Naïve Bayes (MNB), Logistic Regression (LR), and Support Vector Machine (SVM) by applying unigram and n-gram with TF-IDF.

As evident from Table 4, Table 5, Table 6, Table 7, Table 8, Table 9, the comparison results on the $F_{1}$-score considering feature selection with n-grams feature could increase performance in classifying question categories using the traditional models.

**Table 4:** Comparing *F*_1_-score of traditional models (TREC-6 dataset) with focusing POS tags (N+V+Adj+Det+Prep: NVADP).

		Rang of N-gram									
		Micro $F_{1}$-score					Macro $F_{1}$-score				
Dataset/Model		Unigram	**Table:** Uni+ Bigram	**Table:** Uni+ Trigram	Bigram	Trigram	Unigram	**Table:** Uni+ Bigram	**Table:** Uni+ Trigram	Bigram	Trigram
Original	MNB	0.9600	0.9660	0.9660	0.9640	0.9200	0.9538	0.9598	0.9599	0.9676	0.9249
	SVM	0.9640	0.9680	0.9720	0.9580	0.9200	0.9672	0.9709	0.9745	0.9619	0.9239
	LR	0.9600	0.9700	0.9680	0.9460	0.8980	0.9637	0.9728	0.9711	0.9512	0.9044
+all POS Tag	MNB	0.9560	0.9480	0.9380	0.9280	0.8960	0.9159	0.9099	0.9154	0.9058	0.8781
	SVM	**0.9800**	0.9780	0.9760	0.9640	0.9360	**0.9821**	0.9801	0.9780	0.9674	0.9397
	LR	0.9680	0.9740	0.9720	0.9560	0.8920	0.9711	0.9769	0.9741	0.9602	0.9028
+focusing Tags	MNB	0.9560	0.9620	0.9440	0.9400	0.8920	0.9287	0.9468	0.9311	0.9278	0.8720
	SVM	0.9660	0.9760	0.9720	0.9640	0.9280	0.9687	0.9780	0.9747	0.9673	0.9324
	LR	0.9600	0.9640	0.9660	0.9480	0.8780	0.9639	0.9675	0.9690	0.9532	0.8874
+focusing Words	MNB	0.9560	0.9640	0.9640	0.9520	0.9220	0.9503	0.9581	0.9576	0.9551	0.9270
	SVM	0.9680	0.9720	0.9680	0.9640	0.9620	0.9710	0.9747	0.9714	0.9767	0.9278
	LR	0.9520	0.9620	0.9580	0.9460	0.8880	0.9602	0.9658	0.9621	0.9520	0.8923

**Table 5:** Comparing *F*_1_-score of traditional models (TREC-6 dataset) with focusing POS tags (N+V+Adj+Det: NVAD).

		Rang of N-gram									
		Micro $F_{1}$-score					Macro $F_{1}$-score				
Dataset/Model		Unigram	**Table:** Uni+ Bigram	**Table:** Uni+ Trigram	Bigram	Trigram	Unigram	**Table:** Uni+ Bigram	**Table:** Uni+ Trigram	Bigram	Trigram
Original	MNB	0.9600	0.9660	0.9660	0.9640	0.9200	0.9538	0.9598	0.9599	0.9676	0.9249
	SVM	0.9640	0.9680	0.9720	0.9580	0.9200	0.9672	0.9709	0.9745	0.9619	0.9239
	LR	0.9600	0.9700	0.9680	0.9460	0.8980	0.9637	0.9728	0.9711	0.9512	0.9044
+all POS Tag	MNB	0.9560	0.9480	0.9380	0.9280	0.8960	0.9159	0.9099	0.9154	0.9058	0.8781
	SVM	**0.9800**	0.9780	0.9760	0.9640	0.9360	**0.9821**	0.9801	0.9780	0.9674	0.9397
	LR	0.9680	0.9740	0.9720	0.9560	0.8920	0.9711	0.9769	0.9741	0.9602	0.9028
+focusing Tags	MNB	0.9540	0.9620	0.9540	0.9460	0.9040	0.9268	0.9468	0.9391	0.9332	0.8954
	SVM	0.9660	0.9780	0.9700	0.9680	0.9320	0.9687	0.9799	0.9730	0.9708	0.9362
	LR	0.9620	0.9680	0.9620	0.9440	0.8940	0.9656	0.9710	0.9653	0.9496	0.9019
+focusing Words	MNB	0.9560	0.9660	0.9680	0.9500	0.9200	0.9503	0.9600	0.9615	0.9534	0.9251
	SVM	0.9720	0.9720	0.9680	0.9660	0.9300	0.9747	0.9747	0.9712	0.9693	0.9314
	LR	0.9480	0.9580	0.9580	0.9500	0.8880	0.9538	0.9621	0.9621	0.9549	0.8908

**Table 6:** Comparing *F*_1_-score of traditional models (TREC-6 dataset) with focusing POS tags (N+Adj+Det: NAD).

		Rang of N-gram									
		Micro $F_{1}$-score					Macro $F_{1}$-score				
Dataset/Model		Unigram	**Table:** Uni+ Bigram	**Table:** Uni+ Trigram	Bigram	Trigram	Unigram	**Table:** Uni+ Bigram	**Table:** Uni+ Trigram	Bigram	Trigram
Original	MNB	0.9600	0.9660	0.9660	0.9640	0.9200	0.9538	0.9598	0.9599	0.9676	0.9249
	SVM	0.9640	0.9680	0.9720	0.9580	0.9200	0.9672	0.9709	0.9745	0.9619	0.9239
	LR	0.9600	0.9700	0.9680	0.9460	0.8980	0.9637	0.9728	0.9711	0.9512	0.9044
+all POS Tag	MNB	0.9560	0.9480	0.9380	0.9280	0.8960	0.9159	0.9099	0.9154	0.9058	0.8781
	SVM	**0.9800**	0.9780	0.9760	0.9640	0.9360	**0.9821**	0.9801	0.9780	0.9674	0.9397
	LR	0.9680	0.9740	0.9720	0.9560	0.8920	0.9711	0.9769	0.9741	0.9602	0.9028
+focusing Tags	MNB	0.9560	0.9680	0.9600	0.9560	0.9240	0.9395	0.9624	0.9453	0.9414	0.9105
	SVM	0.9680	0.9780	0.9720	0.9640	0.9340	0.9708	0.9799	0.9747	0.9669	0.9377
	LR	0.9620	0.9680	0.9640	0.9520	0.8760	0.9656	0.9712	0.9681	0.9562	0.8841
+focusing Words	MNB	0.9600	0.9740	0.9740	0.9540	0.9180	0.9544	0.9674	0.9672	0.9574	0.9147
	SVM	0.9680	0.9740	0.9720	0.9680	0.9300	0.9710	0.9766	0.9749	0.9709	0.9317
	LR	0.9520	0.9620	0.9620	0.9460	0.8900	0.9564	0.9658	0.9658	0.9526	0.8929

**Table 7:** Comparing *F*_1_-score of traditional models (Thai sentences dataset) with focusing POS tags (N+V+Adj+Det+Prep: NVADP).

		Rang of N-gram									
		Micro $F_{1}$-score					Macro $F_{1}$-score				
Dataset/Model		Unigram	**Table:** Uni+ Bigram	**Table:** Uni+ Trigram	Bigram	Trigram	Unigram	**Table:** Uni+ Bigram	**Table:** Uni+ Trigram	Bigram	Trigram
Original	MNB	0.6425	0.6704	0.6592	0.6648	0.5419	0.6375	0.6726	0.6585	0.6652	0.5344
	SVM	0.7374	0.7318	0.7318	0.6592	0.5363	0.7394	0.7361	0.7331	0.6583	0.5289
	LR	0.7095	0.7039	0.6927	0.6760	0.5307	0.7062	0.7007	0.6898	0.6758	0.5229
+all POS Tags	MNB	0.6760	0.6872	0.6480	0.6927	0.6089	0.6673	0.6827	0.6408	0.6855	0.5959
	SVM	0.7430	0.7374	0.7263	0.7095	0.6201	0.7430	0.7395	0.7253	0.7075	0.6088
	LR	0.7207	0.7318	0.6816	0.6760	0.5866	0.7188	0.7319	0.6741	0.6703	0.5681
+focusing Tags	MNB	0.6704	0.6816	0.6760	0.6816	0.5754	0.6626	0.6763	0.6728	0.6748	0.5634
	SVM	**0.7598**	0.7542	0.7386	0.6872	0.6145	**0.7621**	0.7560	0.7494	0.6867	0.6023
	LR	0.7486	0.7430	0.7374	0.6816	0.5698	0.7469	0.7409	0.7389	0.6794	0.5516
+focusing Words	MNB	0.6648	0.6704	0.6592	0.6536	0.5307	0.6647	0.6723	0.6602	0.6544	0.5260
	SVM	0.7263	0.7095	0.6927	0.6480	0.5307	0.7295	0.7123	0.6957	0.6471	0.5273
	LR	0.6983	0.7039	0.6872	0.6592	0.4972	0.6976	0.7015	0.6844	0.6574	0.4928

**Table 8:** Comparing *F*_1_-score of traditional models (Thai sentences dataset) with focusing POS tags (N+V+Adj+Det: NVAD).

		Rang of N-gram									
		Micro $F_{1}$-score					Macro $F_{1}$-score				
Dataset/Model		Unigram	**Table:** Uni+ Bigram	**Table:** Uni+ Trigram	Bigram	Trigram	Unigram	**Table:** Uni+ Bigram	**Table:** Uni+ Trigram	Bigram	Trigram
Original	MNB	0.6425	0.6704	0.6592	0.6648	0.5419	0.6375	0.6726	0.6585	0.6652	0.5344
	SVM	0.7374	0.7318	0.7318	0.6592	0.5363	0.7394	0.7361	0.7331	0.6583	0.5289
	LR	0.7095	0.7039	0.6927	0.6760	0.5307	0.7062	0.7007	0.6898	0.6758	0.5229
+all POS Tags	MNB	0.6760	0.6872	0.6480	0.6927	0.6089	0.6673	0.6827	0.6408	0.6855	0.5959
	SVM	0.7430	0.7374	0.7263	0.7095	0.6201	0.7430	0.7395	0.7253	0.7075	0.6088
	LR	0.7207	0.7318	0.6816	0.6760	0.5866	0.7188	0.7319	0.6741	0.6703	0.5681
+focusing Tags	MNB	0.6704	0.6872	0.6760	0.6927	0.5866	0.6626	0.6817	0.6697	0.6887	0.5778
	SVM	**0.7654**	0.7486	0.7263	0.6983	0.5866	**0.7685**	0.7502	0.7270	0.7002	0.5741
	LR	0.7598	0.7374	0.7207	0.7039	0.5866	0.7589	0.7372	0.7195	0.7059	0.5763
+focusing Words	MNB	0.6592	0.6592	0.6592	0.6536	0.5196	0.6593	0.6602	0.6592	0.6552	0.5135
	SVM	0.7263	0.6983	0.6816	0.6480	0.5251	0.7297	0.7024	0.6858	0.6485	0.5207
	LR	0.7095	0.6927	0.6704	0.6369	0.5196	0.7083	0.6916	0.6681	0.6369	0.5135

**Table 9:** Comparing *F*_1_-score of traditional models (Thai sentences dataset) with focusing POS tags (N+Adj+Det: NAD).

		Rang of N-gram									
		Micro $F_{1}$-score					Macro $F_{1}$-score				
Dataset/Model		Unigram	**Table:** Uni+ Bigram	**Table:** Uni+ Trigram	Bigram	Trigram	Unigram	**Table:** Uni+ Bigram	**Table:** Uni+ Trigram	Bigram	Trigram
Original	MNB	0.6425	0.6704	0.6592	0.6648	0.5419	0.6375	0.6726	0.6585	0.6652	0.5344
	SVM	0.7374	0.7318	0.7318	0.6592	0.5363	0.7394	0.7361	0.7331	0.6583	0.5289
	LR	0.7095	0.7039	0.6927	0.6760	0.5307	0.7062	0.7007	0.6898	0.6758	0.5229
+all POS Tags	MNB	0.6760	0.6872	0.6480	0.6927	0.6089	0.6673	0.6827	0.6408	0.6855	0.5959
	SVM	0.7430	0.7374	0.7263	0.7095	0.6201	0.7430	0.7395	0.7253	0.7075	0.6088
	LR	0.7207	0.7318	0.6816	0.6760	0.5866	0.7188	0.7319	0.6741	0.6703	0.5681
+focusing Tags	MNB	0.6872	0.6872	0.7039	0.6927	0.6034	0.6809	0.6809	0.6954	0.6885	0.5856
	SVM	0.7374	0.7374	0.7486	0.7263	0.6201	0.7382	0.7382	0.7503	0.7257	0.6055
	LR	**0.7598**	**0.7598**	0.7095	0.6704	0.5866	**0.7599**	**0.7599**	0.7088	0.6695	0.5684
+focusing Words	MNB	0.6318	0.6536	0.6536	0.6425	0.5307	0.6320	0.6541	0.6534	0.6410	0.5247
	SVM	0.6983	0.6760	0.6704	0.6369	0.5307	0.7001	0.6752	0.6688	0.6358	0.5247
	LR	0.6872	0.6760	0.6536	0.6257	0.5251	0.6835	0.6746	0.6505	0.6208	0.5177

We considered different feature sets with focusing POS tags on POS tags (N+V+Adj+Det+Prep) in Table 4, focusing POS tags on POS tags (N+V+Adj+Det) in Table 5, and focusing POS tags on POS tags (N+Adj+Det) in Table 6.

Table 4, Table 5, Table 6 showed the impact of adding contextual word n-grams on the TREC-6 dataset. The results showed that the combined word embedding with contextual word n-grams could improve classification performance. The unigram range had the highest accuracy on the TREC-6 dataset. Besides, the SVM model achieved the micro $F_{1}$-score of 0.98 and macro $F_{1}$-score of 0.9821 when applied unigram and TF-IDF, adding all POS tags. Compared to the unigram baseline on original input, the accuracy of SVM improved from the average micro $F_{1}$-score of 0.9640 to 0.9680 and macro $F_{1}$-score of 0.9672 to 0.9709 (unigram+bigram and TF-IDF). In comparison, the accuracy classifying decreased from 0.9640 to 0.92 for trigram with TF-IDF on original inputs, respectively.

Compared to the results when applied traditional model with the different set of focusing tags baseline on original input. The accuracy of SVM when considered focusing POS tags in a different set of POS tags improved the performance from the original input, but it was not better than adding all POS tags. However, we found that focusing POS tags (N+Adj+Det) and focusing POS tags (N+V+Adj+Det) yielded comparative performance with adding all POS tags with the micro $F_{1}$-score of 0.9780 and macro $F_{1}$-score of 0.9799 when applied on unigram+bigram and TF-IDF.

For the Thai sentences dataset, we considered using different feature sets that are focusing POS tags on POS tags (N+V+Adj+Det+Prep) in Table 7, focusing POS tags on POS tags (N+V+Adj+Det) in Table 8, and focusing POS tags on POS tags (N+Adj+Det) in Table 9.

The results showed that SVM (unigram with TF-IDF), adding focusing POS tag (N+V+Adj+Det) input, achieved the highest average micro $F_{1}$-score of 0.7654 and macro $F_{1}$-score of 0.7685. Compared to the Unigram baseline on the original input, the micro $F_{1}$-score of SVM improved from 0.7374 to 0.7598, both focusing POS tags (N+V+Adj+Det+Prep) and focusing POS tags (N+V+Adj+Det) and improved from 0.7374 to 0.7430 on all POS tags. While the accuracy continuously decreased when applied n-gram with TF-IDF and used SVM model on adding focusing words, as shown in Table 7, Table 8, Table 9.

To study POS tagging enhancement NLP to question classification, we compared the performance of deep learning-based classifiers including CNN, BiLSTM, and Hybrid (CNN with BiLSTM). The experimental results showed that our proposed could increase accuracy question classification, as shown in Table 10. Considering adding POS tags in the data preprocessing method, Table 11 shows the classification performance results on TREC-6. Hybrid model on adding focusing words on focusing POS tag (N+V+Adj+Det) achieved the highest average micro $F_{1}$-score of 0.9750 and average macro $F_{1}$-score of 0.9770. The results of the CNN model applied to add a focusing word on focusing POS (N+Adj+Det) are better results than on original inputs that gave the second-best results with the average micro $F_{1}$-score of 0.9747 and average macro $F_{1}$-score of 0.9767 in Table 12.

**Table 10:** Comparing *F*_1_-score of deep learning models (TREC-6 dataset) with focusing POS tags (N+V+Adj+Det+Prep: NVADP).

Input	Model	Micro-averaged					Macro-averaged				
		*P*	*R*	$F_{1}$(Mean)	$F_{1}$(SD)	$F_{1}$(Max)	*P*	*R*	$F_{1}$(Mean)	$F_{1}$(SD)	$F_{1}$(Max)
Original	CNN	0.9600	0.9600	0.9600	0.0042	0.9680	0.9641	0.9652	0.9641	0.0037	0.9710
	BiLSTM	0.9732	0.9732	0.9732	0.0041	**0.9800**	0.9753	0.9760	0.9755	0.0038	**0.9816**
	Hybrid	0.9718	0.9718	0.9718	0.0027	0.9780	0.9737	0.9746	0.9741	0.0024	0.9796
+all POS Tags	CNN	0.9594	0.9594	0.9594	0.0031	0.9640	0.9628	0.9641	0.9630	0.0029	0.9674
	BiLSTM	0.9753	0.9753	**0.9753**	0.0035	0.9780	0.9772	0.9775	**0.9772**	0.0033	0.9799
	Hybrid	0.9720	0.9720	0.9720	0.0028	0.9760	0.9739	0.9748	0.9742	0.0025	0.9779
+focusing Tags	CNN	0.9630	0.9630	0.9630	0.0041	0.9700	0.9662	0.9678	0.9666	0.0038	0.9727
	BiLSTM	0.9704	0.9704	0.9704	0.0034	0.9760	0.9726	0.9732	0.9727	0.0032	0.9780
	Hybrid	0.9706	0.9706	0.9706	0.0053	0.9760	0.9730	0.9733	0.9729	0.0050	0.9782
+focusing Words	CNN	0.9644	0.9644	0.9644	0.0034	0.9700	0.9672	0.9687	0.9676	0.0031	0.9727
	BiLSTM	0.9738	0.9738	0.9738	0.0020	0.9760	0.9754	0.9764	0.9758	0.0020	0.9784
	Hybrid	0.9734	0.9734	0.9734	0.0035	0.9780	0.9753	0.9764	0.9755	0.0032	0.9797

**Table 11:** Comparing *F*_1_-score of deep learning models (TREC-6 dataset) with focusing POS tags (N+V+Adj+Det: NVAD).

Input	Model	Micro-averaged					Macro-averaged				
		*P*	*R*	$F_{1}$(Mean)	$F_{1}$(SD)	$F_{1}$(Max)	*P*	*R*	$F_{1}$(Mean)	$F_{1}$(SD)	$F_{1}$(Max)
Original	CNN	0.9600	0.9600	0.9600	0.0042	0.9680	0.9641	0.9652	0.9641	0.0037	0.9710
	BiLSTM	0.9732	0.9732	0.9732	0.0041	0.9800	0.9753	0.9760	0.9755	0.0038	0.9816
	Hybrid	0.9718	0.9718	0.9718	0.0027	0.9780	0.9737	0.9746	0.9741	0.0024	0.9796
+all POS Tags	CNN	0.9594	0.9594	0.9594	0.0031	0.9640	0.9628	0.9641	0.9630	0.0029	0.9674
	BiLSTM	0.9753	0.9753	0.9738	0.0035	0.9780	0.9772	0.9775	0.9758	0.0033	0.9799
	Hybrid	0.9720	0.9720	0.9720	0.0028	0.9760	0.9739	0.9748	0.9742	0.0025	0.9779
+focusing Tags	CNN	0.9644	0.9644	0.9644	0.0034	0.9700	0.9675	0.9688	0.9678	0.0029	0.9723
	BiLSTM	0.9738	0.9738	0.9738	0.0033	0.9780	0.9756	0.9764	0.9759	0.0030	0.9799
	Hybrid	0.9694	0.9694	0.9694	0.0035	0.9740	0.9715	0.9729	0.9720	0.0030	0.9760
+focusing Words	CNN	0.9668	0.9668	0.9668	0.0045	0.9740	0.9697	0.9711	0.9699	0.0041	0.9762
	BiLSTM	0.9724	0.9724	0.9724	0.0040	0.9780	0.9745	0.9753	0.9748	0.0035	0.9798
	Hybrid	0.9750	0.9750	**0.9750**	0.0056	**0.9820**	0.9767	0.9779	**0.9770**	0.0051	**0.9834**

**Table 12:** Comparing *F*_1_-score of deep learning models (TREC-6 dataset) with focusing POS tags (N+Adj+Det: NAD).

Input	Model	Micro-averaged					Macro-averaged				
		*P*	*R*	$F_{1}$(Mean)	$F_{1}$(SD)	$F_{1}$(Max)	*P*	*R*	$F_{1}$(Mean)	$F_{1}$(SD)	$F_{1}$(Max)
Original	CNN	0.9600	0.9600	0.9600	0.0042	0.9680	0.9641	0.9652	0.9641	0.0037	0.9710
	BiLSTM	0.9732	0.9732	0.9732	0.0041	0.9800	0.9753	0.9760	0.9755	0.0038	0.9816
	Hybrid	0.9718	0.9718	0.9718	0.0027	0.9780	0.9737	0.9746	0.9741	0.0024	0.9796
+all POS Tags	CNN	0.9594	0.9594	0.9594	0.0031	0.9640	0.9628	0.9641	0.9630	0.0029	0.9674
	BiLSTM	0.9753	0.9753	0.9738	0.0035	0.9780	0.9772	0.9775	0.9758	0.0033	0.9799
	Hybrid	0.9720	0.9720	0.9720	0.0028	0.9760	0.9739	0.9748	0.9742	0.0025	0.9779
+focusing Tags	CNN	0.9618	0.9618	0.9618	0.0047	0.9700	0.9652	0.9666	0.9655	0.0042	0.9732
	BiLSTM	0.9706	0.9706	0.9706	0.0028	0.9760	0.9726	0.9736	0.9729	0.0027	0.9780
	Hybrid	0.9728	0.9728	0.9728	0.0031	0.9780	0.9748	0.9757	0.9751	0.0029	0.9798
+focusing Words	CNN	0.9747	0.9747	**0.9747**	0.0038	0.9780	0.9765	0.9780	**0.9767**	0.0032	0.9795
	BiLSTM	0.9742	0.9742	0.9742	0.0030	0.9780	0.9758	0.9768	0.9761	0.0029	0.9797
	Hybrid	0.9742	0.9742	0.9742	0.0036	**0.9800**	0.9761	0.9772	0.9764	0.0032	**0.9819**

Table 13, Table 14, Table 15 show experimental results on a question classification method for simple sentences into the wh-question category. We found that the CNN model was outstanding. The classification results for questions from the Thai sentences dataset adding a focusing word or POS tag. The CNN-based question classification could improve accuracy by adding focusing POS tags (N+V+Adj+Det) with the average of micro $F_{1}$-score increased up to 0.8 from 0.7793 and average of macro $F_{1}$-score increased up to 0.7975 from 0.7756 on the original input, as shown in Table 14. Moreover, we found that using focusing POS tags (N+V+Adj+Det) when applied CNN model is the best performance with the micro $F_{1}$-score 0.8380 and macro $F_{1}$-score 0.8371.

**Table 13:** Comparing *F*_1_-score of deep learning models (Thai sentences dataset) with focusing POS tags (N+V+Adj+Det+Prep: NVADP).

Input	Model	Micro-averaged					Macro-averaged				
		*P*	*R*	$F_{1}$(Mean)	$F_{1}$(SD)	$F_{1}$(Max)	*P*	*R*	$F_{1}$(Mean)	$F_{1}$(SD)	$F_{1}$(Max)
Original	CNN	0.7793	0.7793	0.7793	0.0119	0.7989	0.7790	0.7759	0.7756	0.0121	0.7962
	BiLSTM	0.7229	0.7229	0.7229	0.0245	0.7542	0.7323	0.7205	0.7183	0.0251	0.7517
	Hybrid	0.7268	0.7268	0.7268	0.0210	0.7542	0.7397	0.7212	0.7231	0.0203	0.7512
+all POS Tags	CNN	0.7888	0.7888	**0.7888**	0.0195	**0.8156**	0.7917	0.7865	**0.7862**	0.0203	**0.8142**
	BiLSTM	0.7523	0.7523	0.7523	0.0357	0.7598	0.7583	0.7475	0.7476	0.0339	0.7592
	Hybrid	0.7467	0.7467	0.7467	0.0312	0.7542	0.7520	0.7528	0.7463	0.0300	0.7522
+focusing Tags	CNN	0.7933	0.7933	0.7933	0.0095	0.8101	0.7957	0.7882	0.7898	0.0093	0.8031
	BiLSTM	0.7536	0.7536	0.7536	0.0260	0.7765	0.7593	0.7502	0.7498	0.0267	0.7759
	Hybrid	0.7408	0.7408	0.7408	0.0262	0.7654	0.7475	0.7378	0.7363	0.0278	0.7644
+focusing Words	CNN	0.7542	0.7542	0.7542	0.0158	0.7765	0.7628	0.7484	0.7502	0.0167	0.7758
	BiLSTM	0.6939	0.6939	0.6939	0.0225	0.7318	0.6964	0.6873	0.6867	0.0245	0.7258
	Hybrid	0.7123	0.7123	0.7123	0.0372	0.7542	0.7139	0.7104	0.7092	0.0360	0.7512

**Table 14:** Comparing *F*_1_-score of deep learning models (Thai sentences dataset) with focusing POS tags (N+V+Adj+Det: NVAD).

Input	Model	Micro-averaged					Macro-averaged				
		*P*	*R*	$F_{1}$(Mean)	$F_{1}$(SD)	$F_{1}$(Max)	*P*	*R*	$F_{1}$(Mean)	$F_{1}$(SD)	$F_{1}$(Max)
Original	CNN	0.7793	0.7793	0.7793	0.0119	0.7989	0.7790	0.7759	0.7756	0.0121	0.7962
	BiLSTM	0.7229	0.7229	0.7229	0.0245	0.7542	0.7323	0.7205	0.7183	0.0251	0.7517
	Hybrid	0.7268	0.7268	0.7268	0.0210	0.7542	0.7397	0.7212	0.7231	0.0203	0.7512
+all POS Tags	CNN	0.7888	0.7888	0.7888	0.0195	0.8156	0.7917	0.7865	0.7862	0.0203	0.8142
	BiLSTM	0.7523	0.7523	0.7523	0.0357	0.7598	0.7583	0.7475	0.7476	0.0339	0.7592
	Hybrid	0.7467	0.7467	0.7467	0.0312	0.7542	0.7520	0.7528	0.7463	0.0300	0.7522
+focusing Tags	CNN	0.8000	0.8000	**0.8000**	0.0158	**0.8380**	0.8018	0.7963	**0.7975**	0.0168	**0.8371**
	BiLSTM	0.7369	0.7369	0.7369	0.0185	0.7598	0.7462	0.7323	0.7319	0.0188	0.7548
	Hybrid	0.7346	0.7346	0.7346	0.0270	0.7877	0.7471	0.7296	0.7305	0.0278	0.7824
+focusing Words	CNN	0.7525	0.7525	0.7525	0.0199	0.7821	0.7563	0.7474	0.7483	0.0201	0.7774
	BiLSTM	0.6810	0.6810	0.6810	0.0276	0.7151	0.6898	0.6762	0.6753	0.0282	0.7101
	Hybrid	0.7156	0.7156	0.7156	0.0283	0.7709	0.7212	0.7100	0.7107	0.0261	0.7660

**Table 15:** Comparing *F*_1_-score of deep learning models (Thai sentences dataset) with focusing POS tags (N+Adj+Det: NAD).

Input	Model	Micro-averaged					Macro-averaged				
		*P*	*R*	$F_{1}$(Mean)	$F_{1}$(SD)	$F_{1}$(Max)	*P*	*R*	$F_{1}$(Mean)	$F_{1}$(SD)	$F_{1}$(Max)
Original	CNN	0.7793	0.7793	0.7793	0.0119	0.7989	0.7790	0.7759	0.7756	0.0121	0.7962
	BiLSTM	0.7229	0.7229	0.7229	0.0245	0.7542	0.7323	0.7205	0.7183	0.0251	0.7517
	Hybrid	0.7268	0.7268	0.7268	0.0210	0.7542	0.7397	0.7212	0.7231	0.0203	0.7512
+all POS Tags	CNN	0.7888	0.7888	0.7888	0.0195	0.8156	0.7917	0.7865	0.7862	0.0203	0.8142
	BiLSTM	0.7523	0.7523	0.7523	0.0357	0.7598	0.7583	0.7475	0.7476	0.0339	0.7592
	Hybrid	0.7467	0.7467	0.7467	0.0312	0.7542	0.7520	0.7528	0.7463	0.0300	0.7522
+focusing Tags	CNN	0.7955	0.7955	**0.7955**	0.0188	**0.8324**	0.7976	0.7929	**0.7932**	0.0189	**0.8309**
	BiLSTM	0.7162	0.7162	0.7162	0.0111	0.7263	0.7212	0.7133	0.7120	0.0119	0.7317
	Hybrid	0.7503	0.7503	0.7503	0.0227	0.7877	0.7540	0.7467	0.7441	0.0208	0.7758
+focusing Words	CNN	0.7313	0.7313	0.7313	0.0107	0.7347	0.7266	0.7271	0.7542	0.0102	0.7471
	BiLSTM	0.6777	0.6777	0.6777	0.0318	0.7207	0.6834	0.6748	0.6740	0.0320	0.7161
	Hybrid	0.7000	0.7000	0.7000	0.0225	0.7207	0.7267	0.7155	0.7191	0.0225	0.7191

**Figure 5:**
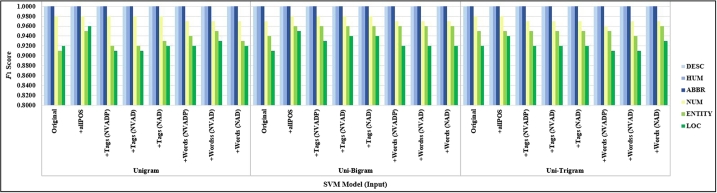
The results of the traditional models (SVM models) grouped by class (TREC-6 dataset).

We evaluate the performance of question classification by comparing an $F_{1}$-score group by question classes between difference input from purposed our data preprocessing tasks on the TREC-6 dataset and Thai sentences dataset. The classification results for TREC-6 yielded the best performance with an $F_{1}$-score of 1.00 for class type “DESC,” “HUM,” and “ABBR” by using both traditional models and deep learning models. There were followed with “NUM,” “ENTITY,” and “LOC” categories, respectively, as shown in Figure 5, Figure 7.

**Figure 6:**
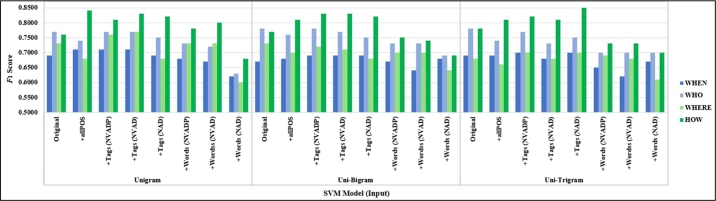
The results of the traditional models (SVM models) grouped by class (Thai sentences dataset).

We measured the performance of the classifiers based on the average $F_{1}$-score group by question classes in the Thai sentences dataset. SVM achieved the highest accuracy for the “HOW” category. Adding focusing POS tags and all POS tags could improve performance for classifying to the “HOW” category, as shown in Fig. 6. Deep learning models obtained the “WHEN” class with the highest average performance on the experiments. Adding focusing and all POS tags could improve performance for classifying to the “HOW” category when applied on BiLSTM and hybrid models. The “WHO” category increased performance by using CNN model with focusing POS tags, as shown in Fig. 8.

**Figure 7:**
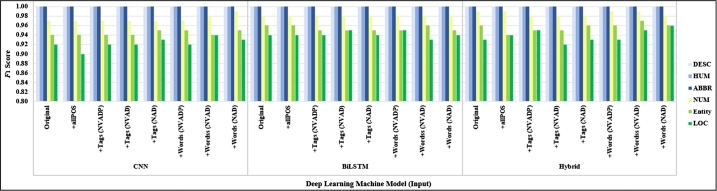
The results of the deep learning models grouped by class (TREC-6 dataset).

**Figure 8:**
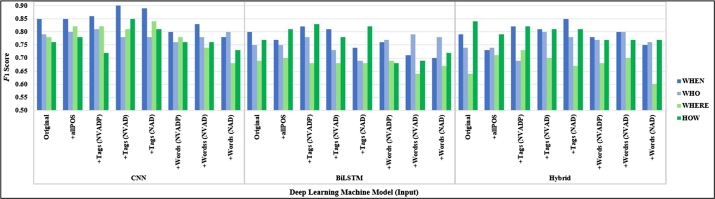
The results of the deep learning models grouped by class (Thai sentences dataset).

## 6 Conclusion and future work

In this paper, we proposed a Part-of-Speech-based feature selection technique for improving the question classification performance. As the current state-of-the-art feature selection approaches work on question classification in English, the Thai language has yet to investigate question classification. The feature selection approaches considering the Part-of-Speech (POS) tag can boost performance for classifying question type from the sentence in Thai texts. We have explored the frequency occurs of POS from the sentence. We considered focusing on POS that could define question types. We experimented by preparing inputs that add words or POS tags as inputs.

To prove the concept of the proposed method, we performed several experiments on both the TREC-6 dataset and the Thai sentences dataset. We used traditional models: Logistic Regression, Multinomial Naïve Bayes, and Support Vector Machine by applied word embeddings, including Unigram, Bigram, and Trigram as N-gram. We also joined Unigram with Bigram and Trigram embeddings with TF-IDF features. Moreover, we investigated the CNN, BiLSTM, and Hybrid model using word embeddings with Global Vectors for Word Representation (GloVe). The experimental results showed that the proposed technique performs significantly better results accuracy on question classification both TREC-6 dataset and Thai sentences dataset when applied considering feature selection. Adding POS tags could increase performance for question classification. The experimental results showed that SVM was a better performance than other traditional classification models. The classifying question categories for a traditional model with micro $F_{1}$-score of 0.98 and macro $F_{1}$-score of 0.9821 when applied Unigram and TF-IDF using SVM model on adding all POS tags in the TREC-6 dataset. The highest micro $F_{1}$-score of 0.9820 and macro $F_{1}$-score of 0.9834 when using the Hybrid model on adding focusing words with focusing POS tags (N+V+Adj+Det).

The experimental results of Thai sentences show that the highest average micro $F_{1}$-score achieved 0.8 using the CNN model, adding focusing POS tags (N+V+Adj+Det) on the Thai sentences dataset. Part-of-Speech tagging was necessary for our proposed method to determine words that the senses can answer and trend to question class. Using only keywords is not enough for classifying simple sentences into Wh-question types. Some words could have many meanings depending on context. Thus, POS tagging could be a benefit for resolve confusion sense for predict question class from the sentence via considering focusing POS tags and POS sequence. A similar state of the art in [16] showed POS tags could aid a model to learn the syntactic function of words. The results we obtained were consistent with the results of [11] combining POS tagging learning can marginally enhance question classification.

Besides, we found that adding too many words or terms acted more like noise rather than improving the performance of the classifiers depending on the dataset. Therefore, focusing on POS tag features can be used for an NLP solution. Especially, Thai sentence analysis requires an efficient method for identifying POS correctly. It requires a high-performance word segmentation because there is no word boundary indicator like the English language, which may cause unwilling POS tagging. Moreover, if we could choose to focus on POS tags or keywords related to the answer could increase the accuracy of classifying questions [13].

Question classification techniques in English and Thai sentences could apply to other applications such as Question Generation that need investigation. Correctly classifying questions considering the feature and related information would affect the possibility of generating the question automated. The limitation of our experiments was no benchmark dataset on question classification in Thai sentences. In future work, we would like to contribute a large dataset for question classification on Thai texts.

## Declarations

### Author contribution statement

Saranlita Chotirat, Phayung Meesad: Conceived and designed the experiments; Performed the experiments; Analyzed and interpreted the data; Contributed reagents, materials, analysis tools or data; Wrote the paper.

### Funding statement

This research received funding from 10.13039/501100016204Ministry of Higher Education, Science, Research and Innovation, Thailand.

### Data availability statement

Data will be made available on request.

### Declaration of interests statement

The authors declare no conflict of interest.

### Additional information

No additional information is available for this paper.
